# Evolution of treatment in gastric cancer- a systematic review

**DOI:** 10.1186/s43046-022-00114-7

**Published:** 2022-03-21

**Authors:** Sangram K. Panda, Pradyumna K. Sahoo, Sunil K. Agarwala, Tim Houghton T, Prathamesh Panchakshari Chandrapattan, Vikas Sankar K, Ramkishan Nag

**Affiliations:** Department of Surgical Oncology, IMS and SUM Hospital, Kalinga Nagar, Bhubaneswar, Odisha India

**Keywords:** Gastric cancer, Treatment, Multimodality, Trials

## Abstract

Multimodality is the standard of care in gastric cancer but surgery remains the mainstay of curative treatment. As we are heading towards a more conservative approach for functional preservation without compromising oncological outcomes in all malignancies, the guidelines keeps changing based on various studies.

The extent of surgery used to vary between the east and west, with the east performing more radical surgery and the west more reliant on multimodality therapy. This practice has been changing in the recent times.

In this article we have reviewed how the treatment protocols of gastric cancer has evolved and modified, highlighting the practice changing trials.

## Main text

### Introduction

Gastric cancer is the fifth most common cancer worldwide and the third most common cause of cancer related death [1]. Countries with high incidence such as South Korea and Japan with a screening program for its citizens has lead to early diagnosis and improved gastric cancer survival, whereas the survival rates in west and in india remains low as most of the cases present in advanced stage.

Multimodality is the standard of care in gastric cancer but surgery remains the mainstay of curative treatment. As we are heading towards a more conservative approach for functional preservation without compromising oncological outcomes in all malignancies, the guidelines keeps changing based on various studies. In this article we have reviewed how the treatment of gastric cancer has evolved and modified highlighting some practice changing trials.

### Surgical management of gastric cancer

In gastric adenocarcinoma limited to the mucosa (T1a), due to the development of imaging techniques such as EUS, and better characterisation in clinical staging, organ preservation can be possible with EMR/ESD, thus avoiding gastrectomy. Sentinel lymph node (SLN) navigation surgery in addressing the regional lymph node is still controversial.

In T1b-T4 tumours, a distal, subtotal or total gastrectomy with adequate lymph node dissection (D2) is the standard practice.

A ***Extent of lymph node dissection***

Adequate nodal dissection of peri gastric lymph nodes and lymph nodes along major vessels is important for staging and prognosticating the disease. In the AJCC 8^th^ edition it is suggested at least 16 regional lymph nodes to be removed for pathological staging but removal of more than 30 lymph nodes is desirable. So different RCT’s studies on the extent of lymph node dissection.

I D1 versus D2 lymphadenectomy:

Both the ***Dutch*** (1995, *n* = 996) [2]and ***MRC*** (1996, *n* = 737) [3] trial, the first western trials comparing D1 and D2 lymphadenectomy, failed to show a survival benefit from D2 lymphadenectomy on 5 year follow up. The participating surgeons were not adequately trained and the post operative complications were significantly high due to higher rates of splenectomy and distal pancreatectomy which was then recommended as a part of D2 lymphadenectomy by the Japanese guidelines except for antral cancers.

However, a 15 year follow up from the Dutch trial showed overall survival was higher in D2 group compared with D1 (29% vs 21%, *p* = 0.34) which was not statistically significant. However, gastric-cancer-related death was significantly higher in the D1 group compared with the D2 group (48% vs 37%, HR 0·74 for D2 *vs* D1, 95% CI 0·59–0·93, *p* = 0·01), Local recurrence was 22% in the D1 group versus 12% in D2, and regional recurrence was 19% in D1 versus 13% in D2 [4].

A subsequent study by the ***Italian**** gastric cancer study group (2010, n* = *267)* showed that D2 lymphadenectomy can be safely performed avoiding splenectomy and distal pancreatectomy in majority of patients [5]

II D1 versus D3 lymphadenectomy:

A single centre RCT from Taiwan (2006, *n* = 335) [6] is the only trial showing survival benefit from an extended lymph node dissection (N1-N3). Five year overall survival was significantly better in D3 group compared to D1 group (59.5% vs 53.6%) difference between groups 5.9%, *p* = 0.04.

III D2 versus PAND lymphadenectomy:

The Japanese RCT by Sasako et al. (***JCOG 9501***, 2009, *n* = 523) [7] proved that an extended lymphadenectomy more than D2 does not add any survival benefit in gastric cancer. The five year overall survival was 70.3% in the D2 lymphadenectomy plus PAND arm, whereas it was 69.2% in arm assigned to D2 lymphadenectomy alone (*p* = 0.85). The recurrence free survival was also not significantly different between the two arms. (*p* = 0.56).

Hence NCCN guidelines recommends that a D2 lymphadenectomy is the standard in curative resections in gastric cancer.

B ***Bursectomy versus no bursectomy***

Bursectomy for distal tumours is routinely practiced by many surgeons worldwide. The meta analysis by **Marano** et al. (2018, n-1340) [8] showed significant overall survival in serosa positive cases with bursectomy, pooled HR = 0.72, 95% CI 0.73–0.99 (*p* < 0.05).

However, the RCT from Japan, ***JCOG 1001*** (*n* = 1503) [9] trial published in 2018 showed that bursectomy does not add a survival benefit in cT3/T4a patients. Five year overall survival was 76.9% in the group assigned to bursectomy and 76.7% in the non-bursectomy group (*p* = 0.65). The burectomy group had a significantly more common incidence of pancratic fistula, 29% vs 15%, *p* = 0.032.

III ***Splenectomy versus no splenectomy***

Splenic hilar nodes(station 10) removal usually requires splenectomy. The RCT by Sano et al. (***JCOG 0110, 2017, n* = *505***) [10] compared routine splenectomy with station 10 removal in total gastrectomy versus no splenectomy. The five year overall survival was 75.1% in splenectomy group and 76.4% in the spleen preservation group. HR was 0.88 (90.7% CI 0.67 – 1.16) (< 1.21); Thus, splenic preservation was proven to be non-inferior to splenectomy (P 1⁄4 0.025). The trial concluded that splenectomy should be avoided unless greater curvature is involved by the tumour or gross nodes are present at station 10.

IV ***Omentectomy versus no omentectomy***

As a part of radical D2 gastrectomy, total omentectomy is done. Few retrospective studies have reported that omentectomy increased post-operative abdominal complications but provided no survival advantage over omentum preservation. ***JCOG1711, ROAD-GC*** trial [11] are ongoing comparing omentectomy versus omental preservation in cT3 and cT4a gastric cancers.

E ***Laparoscopic versus open gastrectomy***

Laparoscopic radical gastrectomy has been proven to have equivalent oncological outcomes with the short term benefits of laparoscopy in both distal and total gastrectomy for early gastric cancers (***KLASS 1, JCOG 0912, KLASS 03***) [12–14].

In locally advanced distal gastric cancer, laparoscopic radical distal gastrectomy is safe and effective as shown by ***CLASS 01, KLASS 02*** trials [15, 16].

But for total gastrectomy the ongoing ***JLSSG0901*** trial will make clear the outcomes and benefits of Laparoscopic procedure in locally advanced gastric cancers (Table 1.).

**Table 1:** Surgical trials

TRIAL / AUTHOR	PURPOSE	RESULT
Dutch, Bonenkamp et al	D1 VS D2 lymphadenectomy	Gastric cancer related death, Locoregional recurrence lower in D2
MRC, A Cushieri et al	D1 VS D2 lymphadenectomy	D2 has no survival benefit over D1
Italian gastric cancer study group, Degiuli et al	D1 VS D2 lymphadenectomy	D2 Lymphadenectomy can be safely performed comparable to D1
Wu et al. (Taiwan)	D1 VS D3 lymphadenectomy	D3 group has better overall survival
JCOG 9501, Fujimura	D2 versus PAND lymphadenectomy	No benefit of PAND
Marano et al	Bursectomy versus no bursectomy	Better Overall survival
JCOG1001, Kurokawa et al	Bursectomy versus no bursectomy	No benefit
JCOG 0110, Sano T et al	Splenectomy versus no splenectomy	No benefit of routine splenectomy
JCOG 1711, ROAD -GC, Sato et al	Omentectomy versus no omentectomy	Ongoing
KLASS 01, CLASS 02, JCOG 0912	Laparoscopy versus Open distal gastrectomy (Early stage)	equivalent oncological outcomes
KLASS03, Hyung et al	Laparoscopy versus Open total gastrectomy (Stage I)	equivalent oncological outcomes
KLASS 02, CLASS 01	Laparoscopy versus Open distal gastrectomy (Locally advanced))	equivalent oncological outcomes
JLSSG0901, katai et al	Laparoscopy versus Open total gastrectomy (Locally advanced)	Ongoing

### Perioperative chemotherapy

Perioperative chemotherapy with ECF 3 cycles before and after surgery is routinely practised based on the ***MAGIC*** trial (2006, *n* = 503) [17] which showed a 13% improvement in 5 year OS which corresponds to a lowering of risk of death by 25%.

Whether the addition of Bevacizumab, a monoclonal antibody against VEGF, has any additional benefit was analysed in the **MRC ST03 trial** [18]. There was no added benefit, but rather complications were higher in the bevacizumab group due to impaired wound healing.

However, the newer recommended regimen now is FLOT, following the results of ***FLOT 4-AIO*** (2019, *n* = 716) trial [19] which showed a significantly better median progression free survival (PFS) compared to ECF regimen (30 vs 18 months, HR: 0.75; 95% CI: 0.62–0.91, *p*: 0.004) and also median overall survival (50 vs 35 months, HR: 0.77; 95% CI: 0.63–0.94, *p*: 0.012). The FLOT regimen also resulted in a higher pathological complete response rate with similar complication rates.

### Adjuvant chemotherapy

If the patient has not received a neoadjuvant chemotherapy, stage II or higher will benefit from adjuvant chemotherapy as shown by the ***ACTS-GC*** trail (2011) [20] where adjuvant S1 was used for 1 year. The five year overall survival improved from 61.1% (95% CI, 56.8% to 65.3%) in the surgery-only group to 71.7% (95% CI, 67.8% to 75.7%) in the S-1 group. The hazard ratio for death in S-1 group in comparison to surgery only group was 0.669 (95% CI, 0.540 to 0.828) which showed that the risk of death was reduced by 33.1% by giving adjuvant S-1. The five year recurrence free survival rate improved from 53.1% to 65.4% with S-1. The Hazard ratio for relapse was 0.653(95% CI, 0.537 to 0.793), showing that S-1 reduced the relapse risk by 34.7%.

In the ***CLASSIC*** trial (2012) [21] adjuvant CAPOX was analysed which showed a 3 year DFS 74% vs 59%, *p*- < 0.0001 (Table 2.).

**Table 2:** Neoadjuvant/ Adjuvant Chemotherapy trials

TRIAL / AUTHOR	PURPOSE	RESULT
MAGIC/ Cunningham et al., 2006	ECF + surgery vs Surgery alone	Improved Overall survival
MRC ST03 trial/ Cunningham et al., 2017	ECF + Bevacizumab vs ECF	No benefit
*FLOT 4-AIO/* Al-Batran et al., 2019	FLOT vs ECF/ECX	Improved Median survival
*ACTS-GC/* Sasako et al., 2011	Surgery + S1 vs Surgery	Improved Overall survival
*CLASSIC/* Bang et al., 2012	Surgery + CAPOX vs Surgery	Better DFS

### Adjuvant chemoradiotherapy

The landmark ***INT -0116*** trial (2000) [22] showed a 3 year OS benefit of 50 vs 41% p-0.0005, and DFS 48 vs 31% with adjuvant chemoradiotherapy. Here, post operative radiation of 45 Gy (1.8 Gy/day, 25#) with bolus FU and LV was used. But this trial was criticised because of inadequate surgery as D0 lymphadenectomy in 54%, D2 lymphadenectomy performed only in 10% of cases and hence the benefit of radiation was likely due to the inadequate surgery.

The subsequent ***ARTIST*** trial (2012) [23] showed no survival benefit after chemoradiation following D2 lymphadenectomy but a subgroup analysis showed DFS benefit only in node positive patients ( 77.5 vs 72.3 p-0.0365) which lead to the ***ARTIST II*** trial (2019) [24] that included only pathological node positive patients following D2 gastrectomy. The Interim results of the ARTIST II trial showed no benefit of adding radiotherapy to chemotherapy even in node positive patients. (HR 0.971; P—0.879).

The ***CRITICS*** trial (2018) [25] also concluded that there is no benefit of in addition of radiotherapy in patients receiving perioperative chemotherapy. The median OS was 43 months (95% CI 31–57) in the chemotherapy arm and 37 months (30–48) in the Chemoradiotherapy arm (hazard ratio from stratified analysis 1·01 (95% CI 0·84–1·22; *p* = 0·90).

NCCN recommends adjuvant chemotherapy after D2 gastrectomy, however post-operative chemoradiation remains treatment of choice for patients with D1 or D0 lymph node dissection.

### Neoadjuvant chemoradiotherapy

The ongoing ***CRTICS II*** [26] and **TOPGEAR** [27] trials are analysing the benefit and feasibility of neoadjuvant chemoradiotherapy in gastric cancer. At present there is no role (Table 3).

**Table 3:** Neoadjuvant/ Adjuvant Chemoradiotherapy

TRIAL / AUTHOR	PURPOSE	RESULT
*INT -0116/* Macdonald, JS et al., 2001	Surgery + FL + RT vs Surgery	Better overall survival
*ARTIST/ Park SH *et al*., 2015*	XP + RT vs XP	No benefit of adding RT except in node positive disease
*CRITICS/ Cats *et al*., 2018*	Pre ECX (EOX) + Post RT + XP vs Peri ECX (EOX)	No benefit of adding RT
*ARTIST-2/ Park SH *et al*., 2019*	SOX + RT + S-1 vs SOX vs S-1	No benefit of Adding adjuvant RT even in node positive patients
CRITICS -2/ Slagter AE et al	DOC vs DOC + RT vs RT (All neoadjuvant)	Ongoing
TOPGEAR/ Leong et al	Pre and Post op ECF vs Pre ECF + RT + Post ECF	Ongoing

## Conclusions

Although the multidisciplinary management of Carcinoma Stomach has evolved over time, the clinical practice varies between the east and west and in between institutions. At present, the recommendations for curative surgery is a total, subtotal or distal gastrectomy with a D2 lymphadenectomy with a goal to examine 16 or greater nodes.

If initial staging is cT2 or higher, any N then perioperative chemotherapy is preferred. If the patient has not received preoperative therapy, then adjuvant chemotherapy for pT2, N + tumours and Adjuvant Chemoradiation for R1 and R2 resection and those who underwent less than a D2 lymph node dissection. Future studies are aimed at combining targeted and immune therapies with cytotoxic chemotherapy.

## Data Availability

Mentioned in References.

## Abbreviations

EUS: Endoscopic ultrasound
EMR: Endoscopic mucosal resection
ESD: Endoscopic submucosal dissection
NCCN: National Comprehensive cancer network
RCT: Radomised control trial
PFS: Progression free survival
OS: Overall survival
RT: Radiotherapy
F: FU- 5 Fluorouracil
LV: Leucovorin
X,C: Capecitabine
P: Paclitaxel
E: Epirubicin
O: OX- Oxaliplatin
s: S1
D: Docetaxel
C: Cyclophosamide

## References

[1] Sung H, Ferlay J, et al. GLOBOCAN Estimates of Incidence and Mortality Worldwide for 36 Cancers in 185 Countries. CA Cancer J Clin. 2021;71(3):209–49. 10.3322/caac.21660. 33538338 10.3322/caac.21660

[2] Bonenkamp J J, Hermans J, et al. Extended Lymph-Node Dissection for Gastric Cancer. N Engl J Med. 1999;340(12):908–14. 10.1056/NEJM199903253401202. 10089184 10.1056/NEJM199903253401202

[3] for the Surgical Co-operative Group, Cuschieri A, Weeden S, et al. Patient Survival after D1 and D2 Resections for Gastric Cancer: Long-Term Results of the MRC Randomized Surgical Trial. Br J Cancer. 1999;79(9–10):1522–30. 10.1038/sj.bjc.6690243. 10188901 10.1038/sj.bjc.6690243 PMC2362742

[4] Songun I, Putter H, et al. Surgical Treatment of Gastric Cancer: 15-Year Follow-up Results of the Randomised Nationwide Dutch D1D2 Trial. Lancet Oncol. 2010;11(5):439–49. 10.1016/S1470-2045(10)70070-X. 20409751 10.1016/S1470-2045(10)70070-X

[5] Degiuli M, Sasako M, Ponti A. Morbidity and Mortality in the Italian Gastric Cancer Study Group Randomized Clinical Trial of D1 *versus* D2 Resection for Gastric Cancer. Br J Surg. 2010;97(5):643–9. 10.1002/bjs.6936. 20186890 10.1002/bjs.6936

[6] Wu C-W, Hsiung CA, et al. Nodal Dissection for Patients with Gastric Cancer: A Randomised Controlled Trial. Lancet Oncol. 2006;7(4):309–15. 10.1016/S1470-2045(06)70623-4. 16574546 10.1016/S1470-2045(06)70623-4

[7] Sasako M, Sano T, Yamamoto S, et al. D2 Lymphadenectomy Alone or with Para-aortic Nodal Dissection for Gastric Cancer. N Engl J Med. 2008;359(5):453–62. 10.1056/nejmoa0707035. 18669424 10.1056/NEJMoa0707035

[8] Marano L, Polom K, et al. Oncologic Effectiveness and Safety of Bursectomy in Patients with Advanced Gastric Cancer: A Systematic Review and Updated Meta-Analysis. J Invest Surg. 2018;31:529–38. 10.1080/08941939.2017.1355942. 28972457 10.1080/08941939.2017.1355942

[9] Kurokawa Y, Doki Y, et al. Bursectomy versus Omentectomy Alone for Resectable Gastric Cancer (JCOG1001): A Phase 3, Open-Label. Randomised Controlled Trial Lancet Gastroenterol Hepatol. 2018;3(7):460–8. 10.1016/S2468-1253(18)30090-6. 29709558 10.1016/S2468-1253(18)30090-6

[10] Sano T, Sasako M, Mizusawa J, et al. Randomized Controlled Trial to Evaluate Splenectomy in Total Gastrectomy for Proximal Gastric Carcinoma. Ann Surg. 2017;265(2):277–83. 10.1097/sla.0000000000001814. 27280511 10.1097/SLA.0000000000001814

[11] Sato Y, Yamada T, et al. Randomized Controlled Phase III Trial to Evaluate Omentum Preserving Gastrectomy for Patients with Advanced Gastric Cancer (JCOG1711, ROAD-GC. Jpn J Clin Oncol. 2020;50(11):1321–4. 10.1093/jjco/hyaa113. 32638017 10.1093/jjco/hyaa113

[12] Kim H-H, Han S-U, et al. Effect of Laparoscopic Distal Gastrectomy vs Open Distal Gastrectomy on Long-Term Survival Among Patients With Stage I Gastric Cancer: The KLASS-01 Randomized Clinical Trial. JAMA Oncol. 2019. 5(4):506-513;5(4):506–13. 10.1001/jamaoncol.2018.6727. 30730546 10.1001/jamaoncol.2018.6727 PMC6459124

[13] Katai H, Mizusawa J, Katayama H, et al. Survival Outcomes after Laparoscopy-Assisted Distal Gastrectomy versus Open Distal Gastrectomy with Nodal Dissection for Clinical Stage IA or IB Gastric Cancer (JCOG0912): A Multicentre, Non-Inferiority, Phase 3 Randomised Controlled Trial. Lancet Gastroenterol Hepatol. 2020. 5(2):142-151;5(2):142–51. 10.1016/S2468-1253(19)30332-2. 31757656 10.1016/S2468-1253(19)30332-2

[14] Hyung W J, Yang H-K, et al. A Feasibility Study of Laparoscopic Total Gastrectomy for Clinical Stage I Gastric Cancer: A Prospective Multi-Center Phase II Clinical Trial, KLASS 03. Gastric Cancer. 2019. 22(1): 214–22;22(1):214–22. 10.1007/s10120-018-0864-4. 30128720 10.1007/s10120-018-0864-4

[15] Yu J, Huang C, et al. Effect of Laparoscopic vs Open Distal Gastrectomy on 3-Year Disease-Free Survival in Patients With Locally Advanced Gastric Cancer: The CLASS-01 Randomized Clinical Trial. JAMA. 2019. 321(20):1983;321(20):1983. 10.1001/jama.2019.5359. 31135850 10.1001/jama.2019.5359 PMC6547120

[16] Hyung W J, Yang H-K, et al. Long-Term Outcomes of Laparoscopic Distal Gastrectomy for Locally Advanced Gastric Cancer: The KLASS-02-RCT Randomized Clinical Trial. J Clin Oncol. 2020;38(28):3304. 10.1200/JCO.20.01210. 32816629 10.1200/JCO.20.01210

[17] Cunningham D, Allum W H, et al. Perioperative Chemotherapy versus Surgery Alone for Resectable Gastroesophageal Cancer. N Engl J Med. 2006;355(1):11–20. 10.1056/NEJMoa055531. 16822992 10.1056/NEJMoa055531

[18] Cunningham D, Stenning SP, Smyth EC, et al. Peri-operative chemotherapy with or without bevacizumab in operable oesophagogastric adenocarcinoma (UK Medical Research Council ST03): primary analysis results of a multicentre, open-label, randomised phase 2–3 trial. Lancet Oncol. 2017;18(3):357–70. 10.1016/S1470-2045(17)30043-8. 28163000 10.1016/S1470-2045(17)30043-8 PMC5337626

[19] Al-Batran S-E, Homann N, et al. Perioperative Chemotherapy with Fluorouracil plus Leucovorin, Oxaliplatin, and Docetaxel versus Fluorouracil or Capecitabine plus Cisplatin and Epirubicin for Locally Advanced, Resectable Gastric or Gastro-Oesophageal Junction Adenocarcinoma (FLOT4): A Randomised, Phase 2/3 Trial. The Lancet. 2019;393(10184):1948–57. 10.1016/S0140-6736(18)32557-1. 10.1016/S0140-6736(18)32557-1 30982686

[20] Sasako M, Sakuramoto S, et al. Five-Year Outcomes of a Randomized Phase III Trial Comparing Adjuvant Chemotherapy with S-1 versus Surgery Alone in Stage II or III Gastric Cancer. *J Clin Oncol: Official J Am Soc Clin Oncol*. 2011;29(33):4387–93. 10.1200/JCO.2011.36.5908. 10.1200/JCO.2011.36.5908 22010012

[21] Bang Y-J, Kim Y-W, et al. Adjuvant Capecitabine and Oxaliplatin for Gastric Cancer after D2 Gastrectomy (CLASSIC): A Phase 3 Open-Label, Randomised Controlled Trial. Lancet (London, England). 2012;379(9813):315–21. 10.1016/S0140-6736(11)61873-4. 22226517 10.1016/S0140-6736(11)61873-4

[22] Macdonald J S, Smalley S R, et al. Chemoradiotherapy after Surgery Compared with Surgery Alone for Adenocarcinoma of the Stomach or Gastroesophageal Junction. N Engl J Med. 2001;345(10):725–30. 10.1056/NEJMoa010187. 11547741 10.1056/NEJMoa010187

[23] Park S H, Sohn T S, et al. Phase III Trial to Compare Adjuvant Chemotherapy With Capecitabine and Cisplatin Versus Concurrent Chemoradiotherapy in Gastric Cancer: Final Report of the Adjuvant Chemoradiotherapy in Stomach Tumors Trial, Including Survival and Subset Analyses. J Clin Oncol: Official J Am Soc Clin Oncol. 2015;33(28):3130–6. 10.1200/JCO.2014.58.3930. 10.1200/JCO.2014.58.3930 25559811

[24] Park S H, Zang D Y, et al. ARTIST 2: Interim Results of a Phase III Trial Involving Adjuvant Chemotherapy and/or Chemoradiotherapy after D2-Gastrectomy in Stage II/III Gastric Cancer (GC). J Clin Oncol. 2019;37(15_suppl):4001. 10.1200/JCO.2019.37.15_suppl.4001.

[25] Cats A, Jansen E P M, et al. Chemotherapy versus Chemoradiotherapy after Surgery and Preoperative Chemotherapy for Resectable Gastric Cancer (CRITICS): An International, Open-Label, Randomised Phase 3 Trial. The Lancet Oncol. 2018;19(5):616–28. 10.1016/S1470-2045(18)30132-3. 29650363 10.1016/S1470-2045(18)30132-3

[26] Slagter A E, Jansen E P M, et al. CRITICS-II: A Multicentre Randomised Phase II Trial of Neo-Adjuvant Chemotherapy Followed by Surgery versus Neo-Adjuvant Chemotherapy and Subsequent Chemoradiotherapy Followed by Surgery versus Neo-Adjuvant Chemoradiotherapy Followed by Surgery in Resectable Gastric Cancer. BMC Cancer. 2018;18(1):877. 10.1186/s12885-018-4770-2. 30200910 10.1186/s12885-018-4770-2 PMC6131797

[27] Leong T, Smithers B M, et al. TOPGEAR: A Randomized, Phase III Trial of Perioperative ECF Chemotherapy with or Without Preoperative Chemoradiation for Resectable Gastric Cancer: Interim Results from an International, Intergroup Trial of the AGITG, TROG, EORTC and CCTG. Ann Surg Oncol. 2017;24(8):2252–8. 10.1245/s10434-017-5830-6. 28337660 10.1245/s10434-017-5830-6

